# Is Antithrombin Supplementation Effective in Suppressing ECMO Circuit Thrombosis in Sepsis?

**DOI:** 10.14789/ejmj.JMJ25-0027-R

**Published:** 2025-10-10

**Authors:** JERROLD H. LEVY, NAO UMEI, YUTAKA KONDO, MICHIO MINESHIMA, TOSHIAKI IBA

**Affiliations:** 1 Department of Anesthesiology, Critical Care, and Surgery, Duke University School of Medicine, Durham, NC, USA; 1 Department of Anesthesiology, Critical Care, and Surgery, Duke University School of Medicine, Durham, NC, USA; 2 Department of Emergency and Disaster Medicine, Juntendo University Graduate School of Medicine, Tokyo, Japan; 2 Department of Emergency and Disaster Medicine, Juntendo University Graduate School of Medicine, Tokyo, Japan; 3 Faculty of Medical Science, Juntendo University, Chiba, Japan; 3 Faculty of Medical Science, Juntendo University, Chiba, Japan

**Keywords:** extracorporeal membrane oxygenation, antithrombin, sepsis, thrombosis, coagulation

## Abstract

Extracorporeal membrane oxygenation (ECMO) is increasingly used for the management of severe sepsis-induced cardiopulmonary failure. Despite standard heparin anticoagulation, circuit thrombosis remains a significant complication. Antithrombin (AT), a critical serine protease inhibitor and cofactor for heparin, is often low in septic patients, especially during ECMO, raising concerns about the efficacy of heparin-based anticoagulation. This review explores the role of AT supplementation to suppress ECMO circuit thrombosis in sepsis, integrating epidemiological data, underlying pathophysiology, preclinical research, and clinical evidence. While theoretical rationale and some observational data support the supplementation of AT, definitive clinical trials are lacking. We conclude by outlining future perspectives and research needs to clarify the role of AT in ECMO management for septic patients.

## Introduction

Sepsis, a global health burden that is responsible for millions of deaths annually is characterized by a dysregulated host response to infection leading to life-threatening organ dysfunction. In severe cases where conventional treatments fail, extracorporeal membrane oxygenation (ECMO) may be used to provide temporary cardiopulmonary support. ECMO is lifesaving in refractory sepsis-induced acute lung injury/acute respiratory distress syndrome (ARDS) but introduces complex challenges, notably coagulation disorders and thrombotic events within the ECMO circuit^1, 2)^.

The mainstay of anticoagulation management during ECMO is unfractionated heparin (UFH), which requires antithrombin (AT) for its efficacy. However, septic patients have a marked reduction of AT activity due to consumption, extravascular leakage, impaired synthesis, and protease degradation^3, 4)^. The resulting AT deficiency may lead to heparin resistance, a state where standard doses of UFH fail to achieve therapeutic anticoagulation^5, 6)^.

Thrombosis in ECMO circuits contributes to oxygenator failure, circuit exchange, and systemic embolic events such as stroke and pulmonary embolism^7)^. Recent registry studies and case series have highlighted the frequent need for ECMO circuit interventions in septic patients with low AT levels^8, 9)^. Although AT supplementation is a theoretical approach to restoring anticoagulant activity and suppressing thrombin generation, its efficacy and safety remain controversial, particularly in light of concerns regarding bleeding risk and cost-effectiveness^2, 10-13)^.

This review examines the role of AT supplementation in preventing ECMO circuit thrombosis in septic patients. By integrating data from epidemiologic studies, pathophysiological mechanisms, preclinical experiments, and clinical outcomes, we aim to provide a comprehensive understanding of the current evidence and identify areas for future research.

## Pathophysiology

Sepsis induces a profound alteration in the coagulation cascade, leading to a hypercoagulable state marked by increased thrombin generation, impaired fibrinolysis, and endothelial dysfunction. During sepsis, proinflammatory cytokines such as TNF-α and IL-6 promote tissue factor expression on monocytes and endothelial cells, initiating extrinsic coagulation pathways^14)^. This process is further amplified by decreased levels of natural anticoagulants, including AT, protein C, and tissue factor pathway inhibitor (TFPI)^15)^.

AT plays a central role in inhibiting thrombin and factor Xa, and its activity is essential for the efficacy of unfractionated heparin (UFH). However, during sepsis, AT levels drop significantly due to increased consumption, decreased hepatic synthesis, and degradation by neutrophil elastase and other proteases^16)^. This deficiency not only compromises heparin function but also contributes to uncontrolled thrombin generation and microvascular thrombosis, exacerbating organ dysfunction^17)^. Meanwhile, AT supplementation is expected to suppress the inflammatory response^18)^.

**Figure 1:**
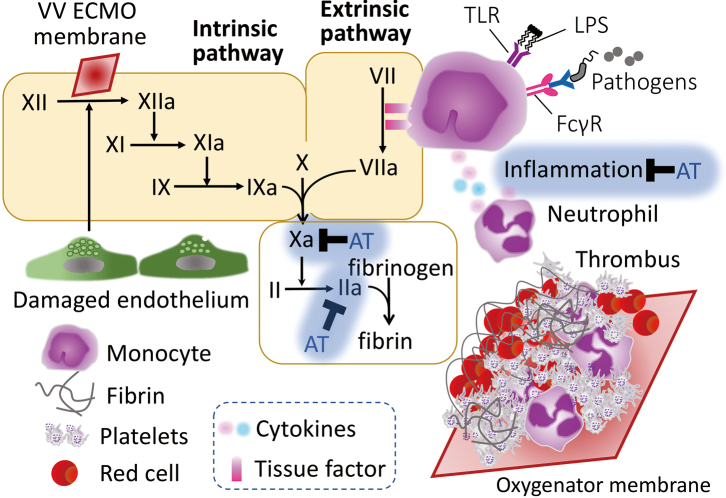
Thrombus formation in the ECMO oxygenator membrane Circulating blood components, including red blood cells, platelets, fibrin, and leukocytes, interact with the non-endothelial surfaces of the extracorporeal membrane oxygenation (ECMO) membrane/circuit. The contact of blood with the ECMO oxygenator membrane surface triggers activation of both intrinsic coagulation cascades. Neutrophils and monocytes contribute to this process through inflammatory signaling pathways, such as tolllike receptor (TLR) activation and Fcγ receptor engagement, promoting further thrombin generation and fibrin deposition. The accumulation of cellular and fibrin components leads to thrombus formation, which can compromise filter function and circuit longevity. Antithrombin and other natural anticoagulants may be consumed in this process, further amplifying procoagulant activity.

The initiation of ECMO further complicates the coagulation status. Blood exposure interfacing with the non-endothelialized ECMO circuit activates contact and complement pathways, leading to platelet activation and further consumption of AT^19)^. In particular, the formation of soluble fibrin and thrombin-antithrombin complexes (TAT) increases, indicating ongoing thrombin generation despite systemic anticoagulation^20)^. ECMO-induced hemolysis and shear stress also stimulate endothelial cell activation and von Willebrand factor release, promoting platelet adhesion and aggregation^21)^ (Figure 1).

Studies have shown that AT has additional anti- inflammatory effects, including inhibition of leukocyte adhesion and endothelial activation, through interaction with syndecans^22)^. These effects may attenuate the endothelial injury seen in both sepsis and ECMO, reducing the risk of thrombosis and capillary leak syndrome.

Overall, the interplay between sepsis-induced coagulopathy and ECMO-associated hemotrauma creates a consumptive coagulopathy state where AT deficiency is a pivotal element. Understanding these mechanisms supports the rationale for investigating AT supplementation as a strategy to stabilize coagulation and improve circuit patency.

## Epidemiology

The incidence of thrombotic complications in patients receiving ECMO for sepsis remains high despite advancements in anticoagulation strategies. Thrombosis of the ECMO circuit components, including the oxygenator, cannula, and pump head, has been reported in 20-40% of adult patients, with some studies suggesting even higher rates in septic populations^23, 24)^. Sepsis induces a prothrombotic state via systemic inflammation, endothelial injury, and activation of the coagulation cascade, predisposing patients to both microvascular thrombosis and macrovascular events^25, 26)^.

Antithrombin deficiency is particularly prevalent in septic patients, with reports indicating that 40-60% of individuals requiring ECMO support have AT activity levels below 60%, and a significant portion of these fall below 50% during the ECMO course^27, 28)^. This deficiency correlates with heparin resistance, a condition in which escalating doses of heparin are required to achieve therapeutic anticoagulation, often without achieving adequate anti-Xa activity^29, 30)^.

Observational studies have demonstrated an association between low AT levels and increased incidence of circuit thrombosis and the need for oxygenator exchange^31, 32)^. Registry data from the Extracorporeal Life Support Organization (ELSO) also identifies thrombotic complications as one of the major causes of morbidity in ECMO, particularly in adults with sepsis-associated coagulopathy^33)^. In addition to mechanical thrombosis, systemic embolic complications such as thromboembolism (VTE) and ischemic stroke further contribute to adverse outcomes^34)^. Especially, an ischemic stroke is a leading cause of mortality in ECMO patients, and the incidence rates range from 1% to 8% and the mortality rates are between 44% and 76%^35)^.

Current anticoagulation strategies vary widely among institutions. While UFH remains the most commonly used agent, its dependency on AT makes AT deficiency a critical modifiable risk factor^36)^. Targeted replacement of AT in cases of documented deficiency has been practiced in some centers to mitigate the thrombotic risk, though data from randomized controlled trials remain lacking^2, 37, 38)^.

In summary, ECMO-supported septic patients represent a high-risk group for thrombotic complications, which are exacerbated by AT deficiency. Understanding the epidemiological burden is essential for designing future interventional trials and for tailoring anticoagulation strategies to individual patient profiles.

## Preclinical studies

Animal and *in vitro* models may provide important mechanistic insights into the role of AT supplementation in the setting of ECMO and sepsis. These preclinical studies will suggest that AT deficiency not only compromises anticoagulant efficacy but also contributes to inflammation-driven endothelial damage, thereby promoting thrombogenesis within ECMO circuits. However, there are a few preclinical studies, probably because it is difficult to reproduce the event in humans^39, 40)^.

In septic animal models, AT supplementation has demonstrated protective effects on vascular integrity and organ function. For instance, in a murine model of endotoxemia, recombinant AT administration reduced systemic inflammation, leukocyte- endothelial interactions, and fibrin deposition in multiple organs^41)^. Similar findings were reported in experimental models of sepsis, where AT supplementation attenuated microvascular thrombosis and reduced oxygenator clot burden^42, 43)^.

In vitro studies using human plasma and endothelial cell cultures have confirmed the biochemical rationale for AT supplementation. AT enhances heparin’s inhibitory effect on thrombin and factor Xa by over 1000-fold, and this effect is severely blunted when AT levels fall below 60%^44, 45)^. Furthermore, AT was shown to reduce nuclear factor-kappa B (NF-κB) activation and intercellular adhesion molecule-1 (ICAM-1) expression on endothelial cells, suggesting that its anti-inflammatory actions may complement its anticoagulant properties^46, 47)^.

Other studies highlight that AT administration may reduce platelet consumption and microthrombus formation during ECMO. In an animal model of veno-arterial ECMO, AT supplementation significantly prolonged circuit lifespan and reduced clot formation within the oxygenator compared to controls^48)^. Despite these promising results, it is important to note the limitations of preclinical studies, including species differences, variable ECMO protocols, and the lack of septic comorbidities, which is a major drawback of the current situation (Table 1).

**Table 1:** Summary of preclinical studies on antithrombin supplementation

Study (Author, Year)	Model used	Intervention	Key findings	Limitations
Dickneite (1998)^4)^	Animal model of sepsis	Antithrombin	Improved survival and reduced organ damage	Non-human model; not ECMO-specific
Buchtele et al. (2021)^10)^	Ex vivo ECMO model	AT vs. placebo	Reduced thrombin generation, improved clotting parameters	In vitro model only
Panigada et al. (2022)^18)^	Human ECMO patients (observational with mechanistic study)	AT administration	Reduced IL-6 and TNF-α; suggested anti-inflammatory effect	Observational; inflammation as surrogate
Schlömmer et al. (2021)^41)^	Cellular/molecular inflammatory model	AT in endotoxemia	Inhibited cytokine production and leukocyte adhesion; endothelial protective effects	Not ECMO; mechanistic only
Rehberg et al. (2013)^43)^	Acute lung injury in animal model	AT	Attenuated vascular leakage and reduced neutrophil activation	No ECMO circuit used
Priest et al. (2025)^48)^	Animal ECMO	AT supplementation	Reduced clot formation in oxygenator, prolonged circuit lifespan	Small sample size; animal model limitations

ECMO: Extracorporeal membrane oxygenation, AT: antithrombin, trial, TNF: tumor necrosis factor, IL: interleukin

## Clinical evidence

Clinical evidence evaluating the role of AT supplementation in ECMO-supported septic patients remains limited and largely observational. Nevertheless, several studies have suggested potential benefits in reducing thrombotic complications and improving circuit patency. Vorisek et al.^49)^ examined the effects of combined AT and heparin therapy and reported superior anticoagulation with less bleeding than high-dose heparin alone, underscoring its potential for hemostatic balance.

Although the accumulating evidence supports the biological plausibility of AT supplementation as a strategy to reduce ECMO-associated thrombosis in sepsis, there is no direct evidence supporting the use of antithrombin^50)^. Retrospective analyses from large ECMO registries and single-center cohorts have reported associations between low AT levels and increased incidence of VTE oxygenator thrombosis and circuit change-outs^51, 52)^. These findings have driven interest in evaluating AT as a therapeutic adjunct in anticoagulation protocols.

A prospective randomized controlled trial (RCT) assessed AT levels in patients with septic shock undergoing ECMO and found that those with AT activity < 60% had a significantly higher rate of clotting complications and required more frequent oxygenator changes^37)^. A single-center, retrospective cohort study of adult ECMO patients reported an association between higher mean AT activity and better ICU survival^53)^. Meanwhile, some showed lower thrombosis but higher mortality with antithrombin supplementation, others showed no mortality effect but higher rates of thrombotic or hemorrhagic events and longer hospital stays with AT^54, 55)^.

Prospective pilot studies have begun to evaluate the safety of AT replacement in ECMO patients. A study by Panigada et al.^18)^ demonstrated that AT supplementation restored AT levels to normal and improved heparin sensitivity without increasing major bleeding events (Table 2).

Nonetheless, concerns remain regarding cost-effectiveness and bleeding risk^56)^. AT concentrates are expensive, and in some studies, their administration has been associated with increased bleeding, particularly when high-dose regimens are used without careful monitoring^57, 58)^. Furthermore, the optimal dosing strategy, timing, and patient selection criteria for AT replacement have not been standardized.

Taken together, the current clinical evidence suggests a promising but not yet definitive role for AT supplementation in septic patients undergoing ECMO^54)^. High-quality, prospective RCTs are urgently needed to determine whether AT improves outcomes such as circuit longevity, thrombotic event reduction, and overall survival without increasing adverse effects.

**Table 2:** Summary of clinical evidence on antithrombin supplementation in ECMO patients

Study (Author, Year)	Study design	Population/Setting	Intervention	Key findings	Limitation
Chlebow ski et al. (2020)^2)^	Expert review	Adult ECMO patients	Literature	Emphasized variability in AT use; raised safety/efficacy concerns	No original data
Panigada et al. (2022)^18)^	Ancillary observational study (GATRA trial)	VV-ECMO patients	AT administration	Decreased IL-6, TNF-α; improved inflammatory profile	Inflammation markers only; non-RCT
Panigada et al. (2020)^37)^	RCT	ECMO with AT deficiency patients	AT vs. control	Designed to evaluate anticoagulation stability and bleeding/thrombotic risk	AT may not decrease heparin nor diminish bleeding and thrombosis
Piacente et al. (2020)^38)^	Narrative review	ECMO patients	No intervention	No consensus on threshold, dose, and time of administration.	General opinion only
Vorisek et al. (2019)^49)^	Retrospective analysis	Pediatric post-cardiac ECMO patients	High-dose AT + heparin	Superior anticoagulation vs. heparin alone; less bleeding	Focused on pediatrics; mixed interventions
Byrnes et al. (2014)^50)^	Retrospective analysis	Pediatric ECMO patients	AT supplementation	Decreased heparin dose requirements and prolonged circuit life	Small sample; retrospective observational
Ranucci et al. (2015)^58)^	TEG-based observational study	Adult ECMO patients	AT measured	Procoagulant pattern observed; AT suggested as contributory	No intervention trial

ECMO: Extracorporeal membrane oxygenation, AT: antithrombin, VV: veno-venous, RCT: randomized controlled

## Future considerations

Based on our review, there is a need for high-quality clinical trials to evaluate the efficacy of AT supplementation in ECMO-supported septic patients with ARDS. The current body of evidence remains largely observational and prone to confounding by indication and center-level variability in practice. To overcome these limitations, as performed in COVID-19, well-designed multicenter RCTs are needed. Such trials should stratify patients by baseline AT levels, clinical considerations of ARDS and indications for veno-venous ECMO, and severity of sepsis to identify populations most likely to benefit^59-61)^.

Technological advances in coagulation monitoring, including point-of-care viscoelastic assays and anti-Xa activity measurements, may enable real-time assessments of heparin responsiveness and help guide targeted AT supplementation^62, 63)^. Biomarker- driven anticoagulation protocols incorporating AT activity thresholds could offer a precision medicine approach to minimizing thrombosis while limiting bleeding risk^64, 65)^. Moreover, the broader role of AT beyond anticoagulation, such as its anti-inflammatory and endothelial-protective effects, warrants further mechanistic exploration^66, 67)^. From a health economics perspective, rigorous cost-effectiveness analyses are needed to justify the routine use of AT concentrates in ECMO, especially given their high expense and limited availability in some settings. Implementation science should also be employed to evaluate how best to integrate AT and heparin monitoring and supplementation into existing ECMO protocols without introducing undue complexity or delay^68, 69)^.

Finally, future studies should consider long-term outcomes, including ECMO weaning success, organ recovery, and quality of life after ICU discharge. By integrating clinical, molecular, and economic data, a comprehensive evaluation of AT supplementation in this high-risk population can be achieved.

## Conclusion

AT supplementation represents a biologically plausible and mechanistically supported intervention to address the prothrombotic state observed in septic patients receiving ECMO. Some clinical studies demonstrate its potential to enhance heparin efficacy, reduce inflammation, and protect the endothelium^70, 71)^. Other evidence, though promising, remains limited to observational studies and small-scale trials, with considerable heterogeneity in patient populations and dosing protocols^72, 73)^.

While AT deficiency is common in ECMO-supported sepsis and has been associated with thrombotic complications, the benefits of routine supplementation have yet to be confirmed in RCTs. Concerns regarding bleeding risk, cost, and standardization of care remain significant barriers to widespread adoption. Nonetheless, the existing data support further investigation of AT replacement, particularly in patients with documented AT deficiency and evidence of heparin resistance.

In conclusion, AT supplementation holds potential as a targeted adjunct therapy in the management of ECMO circuit thrombosis in sepsis. Future randomized trials, mechanistic studies, and cost- benefit analyses will be essential to define its role and to develop optimized protocols that balance efficacy with safety.

## Author contributions

JHL and TI wrote the manuscript. NU, YK and MM revised the text. All authors read and approved the final manuscript.

## Conflicts of interest statement

J. H. Levy and T. Iba, members of the JMJ Editorial Board, were not involved in the peer review or decision-making process for this paper. The authors declare that they have no conflict of interest.
